# Liposome‐based in situ antigen‐modification strategy for “universal” T‐cell‐receptor engineered T cell in cancer immunotherapy

**DOI:** 10.1002/mco2.618

**Published:** 2024-07-07

**Authors:** Qin Wang, Rui Peng, Haoyue Qi, Ruihan Xu, Wanmin Liu, Fanyan Meng, Shiyao Du, Lixia Yu, Jia Wei, Fangcen Liu, Rutian Li

**Affiliations:** ^1^ The Comprehensive Cancer Centre of Nanjing Drum Tower Hospital The Affiliated Hospital of Nanjing University Medical School Nanjing China; ^2^ Department of General Surgery The Affiliated Cancer Hospital of Nanjing Medical University & Jiangsu Cancer Hospital & Jiangsu Institute of Cancer Research Jiangsu China; ^3^ School of Medical Nanjing University Nanjing China; ^4^ Department of Laboratory Medicine Nanjing Drum Tower Hospital Clinical College of Nanjing Medical University Nanjing China; ^5^ Department of Pathology Nanjing Drum Tower Hospital The Affiliated Hospital of Nanjing University Medical School Nanjing China

**Keywords:** anti‐PD‐1 antibody, gastric cancer, liposome, NY‐ESO‐1 antigen delivery, NY‐ESO‐1 T‐cell receptor (TCR) engineered T‐cell therapy

## Abstract

T‐cell receptor (TCR) engineered T‐cell therapy, unlike chimeric antigen receptor T‐cell therapy, relies on the inherent ability of TCRs to detect a wider variety of antigenic epitopes, such as protein fragments found internally or externally on cells. Hence, TCR‐T‐cell therapy offers broader possibilities for treating solid tumors. However, because of the complicated process of identifying specific antigenic peptides, their clinical application still encounters significant challenges. Thus, we aimed to establish a novel “universal” TCR‐T “artificial antigen expression” technique that involves the delivery of the antigen to tumor cells using DSPE‐PEG‐NY‐ESO‐1^157‐165^ liposomes (NY‐ESO‐1 Lips) to express TCR‐T‐cell‐specific recognition targets. In vitro as well as in vivo studies revealed that they could accumulate efficiently in the tumor area and deliver target antigens to activate the tumor‐specific cytotoxic T‐cell immune response. NY‐ESO‐1 TCR‐T therapy, when used in combination, dramatically curbed tumor progression and extended the longevity of mice. Additionally, PD‐1 blockage enhanced the therapeutic effect of the aforementioned therapy. In conclusion, NY‐ESO‐1 Lips “cursed” tumor cells by enabling antigenic target expression on their surface. This innovative technique presents a groundbreaking approach for the widespread utilization of TCR‐T in solid tumor treatment.

## Graphical abstract

NY‐ESO‐1 Lips delivers the antigen to tumor cells in order to “arm” the tumor. Then, NY‐ESO‐1 TCR‐T cells specifically recognize the antigen and induce antitumor effect, resulting in death of cancer cell. Additionally, anti‐PD‐1 antibody can enhance the therapeutic effect.

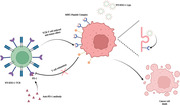

## 1 INTRODUCTION

Recently, T‐cell immunotherapy, grounded on the premise that immune cells can identify and eradicate malignant cells, has stood at the vanguard of cancer treatment. Two primary forms of T‐cell immunotherapies exist: chimeric antigen receptors (CARs) and T cell receptors (TCRs). The effectiveness of the former in treating blood tumors is significant, with multiple CAR products being approved by the US Food and Drug Administration (FDA). Yet, its effectiveness against solid tumors is restricted by various challenges, such as surface protein limitations, inadequate expansion and longevity, and the suppressive environment within tumors.^1^, ^2^ As a backup, T cells that target tumors can examine peptide–human leukocyte antigen (HLA) complexes on the tumor's surface using TCRs. They detected and destroyed tumor cells with abnormal protein expression resulting from intracellular pathogens or cellular transformations, including neoantigens derived for mutations or cancer testis antigens (CTAs), which are mainly intracellularly expressed.^3^ However, it is not possible to use these natural antitumor cells clinically because they are limited, difficult to isolate, and expand on a large scale in vitro. However, when TCR‐coding genes in tumor‐reactive T cells are isolated and transformed into homogeneous T cells, they can be bristled with a tumor‐specific killing ability. Consequently, a substantial quantity of T cells that are specific to antigens can be promptly generated to fulfill the demands of the application. The advantages of these adoptive T‐cell therapy include a wide range of target antigens, high affinity, and increased sensitivity. Therefore, utilizing TCR engineering in T‐cell therapy presents a different method for treating solid tumors with immunotherapy.

Compelling clinical data have been published on TCR‐T‐cell therapies for melanoma, sarcoma, and HPV‐associated epithelial cancers. An enhanced NY‐ESO‐1‐TCR T‐cell therapy showed positive results in treating metastatic melanoma and synovial sarcoma, achieving clinical response rate ranging from 45% to 55%^4^, ^5^ and 50% to 61%,^6^ respectively. Individuals diagnosed with multiple myeloma who underwent the same treatment regimen achieved a clinical response rate of 80%, with noteworthy 70% achieving complete response, and a 19 months median progression‐free survival time.^7^ The phase I clinical trial of HPV‐16 E7 TCR‐T‐cell therapy yielded a 50% clinical response rate among patients suffering from metastatic HPV‐associated epithelial malignancies, including some who were resistant to anti‐PD‐1 antibody.^8^ Additionally, in a different trial (NCT00910650), 69% of patients suffering from metastatic melanoma exhibited positive indications of tumor regression after undergoing treatment with TCR‐T cells targeting MART‐1.

Despite its ideal effectiveness as a cancer immunotherapy, TCR‐T‐cell therapy still faces obstacles that restrict its application. The key to improve TCR‐T‐cell therapy is to identify highly specific target antigens. Currently, the promising TCR‐targeting antigens mainly include neoantigen, CTAs, and virus‐associated antigen. The vast majority of neoantigens in tumors are unique to a given patient or are present at certain time points. Additionally, gene mutations produce antigenic peptides with fairly weak immunogenicity, making the isolation of high‐affinity TCRs difficult. Thus, a treatment focused on the identification of neoantigens is a truly personal therapy and must be customized for each patient,^9^ which is time and cost consuming. CTAs and virus‐related antigens are predominantly present in a small number of tumors, leading to positive outcomes for only a select group of patients receiving targeted TCR‐T therapy.^10^, ^11^, ^12^ Furthermore, the variability in antigen expression within a patient's tumor can lead to resistance to TCR‐T‐cell treatment. Therefore, instead of focusing on identifying universal or personalized antigens, we attempted to construct a “universal” TCR‐T‐cell therapy.

Nobuoka's group^13^ injected ovalbumin antigen peptides into tumor cells' major histocompatibility complex (MHC) I molecule, demonstrating that tumor antigenicity can be altered via an external route. However, there are several limitations: restrictions by the location of tumors, which should be detected by imaging modalities and approached by needles, and difficulty in spreading peptides throughout the tumor. Based on this, we established a novel idea for our “universal” TCR‐T‐cell therapy. To overcome the difficulty of identifying tumor‐specific targets for TCR‐T cells, we attempted to deliver a widely studied tumor‐specific antigen to the tumor surface that can be identified and eliminated by certain TCR‐T cells. Herein, NY‐ESO‐1, a CTA with restricted expression on cancer and testis tissues, instead of normal tissues,^14^ was choose as an exogenous antigen. It has been proven safe in numerous studies and entered in a phase III clinical trial (NCT01343043). Regarding the delivery system, nanoparticles (NPs) have been found as good candidates, which are often used to control drug delivery.^15^, ^16^ Tumor cells uptake them via receptor‐mediated endocytosis.^17^, ^18^ In the realm of cancer immunotherapy, liposomes have emerged as a particularly fitting option due to their resemblance to the cell membrane, offering high biocompatibility, ample surface area, and a non‐cytotoxic nature.^19^ So far, liposomes are usually used to construct tumor vaccine. Irvine et al. have synthesized structurally optimized CpG‐DNA/peptide amph vaccines and demonstrated their superior treatment efficacy as lymph node (LN)‐targeting molecular vaccines.^20^ In our study, we first used liposomes to systemically delivery antigens to tumor cells through their membrane fusion capacity, which developed a new way for liposomes application in “universal” immunotherapy.

In our research, we developed DSPE‐PEG‐NY‐ESO‐1 liposomes (NY‐ESO‐1 Lips) to load the exogenous antigen, NY‐ESO‐1, onto tumor cell membrane. Confocal microscopy and flow cytometry assay demonstrated that NY‐ESO‐1 Lips could bind to antigen deletion variant (ALVs) cells through membrane insertion and express expected MHC–peptide complex on tumor cell surface. NY‐ESO‐1 TCR‐T cells can identify altered ALVs and trigger targeted killing responses in both laboratory settings and living organisms. Thus, solid tumors can be suppressed using this innovative TCR‐T system, even if they do not show NY‐ESO‐1 expression, thanks to the in situ antigen modification approach we developed.

## 2 RESULTS

### 2.1 Characterization of the DSPE‐PEG‐NY‐ESO‐1 conjugate

**FIGURE 1:**
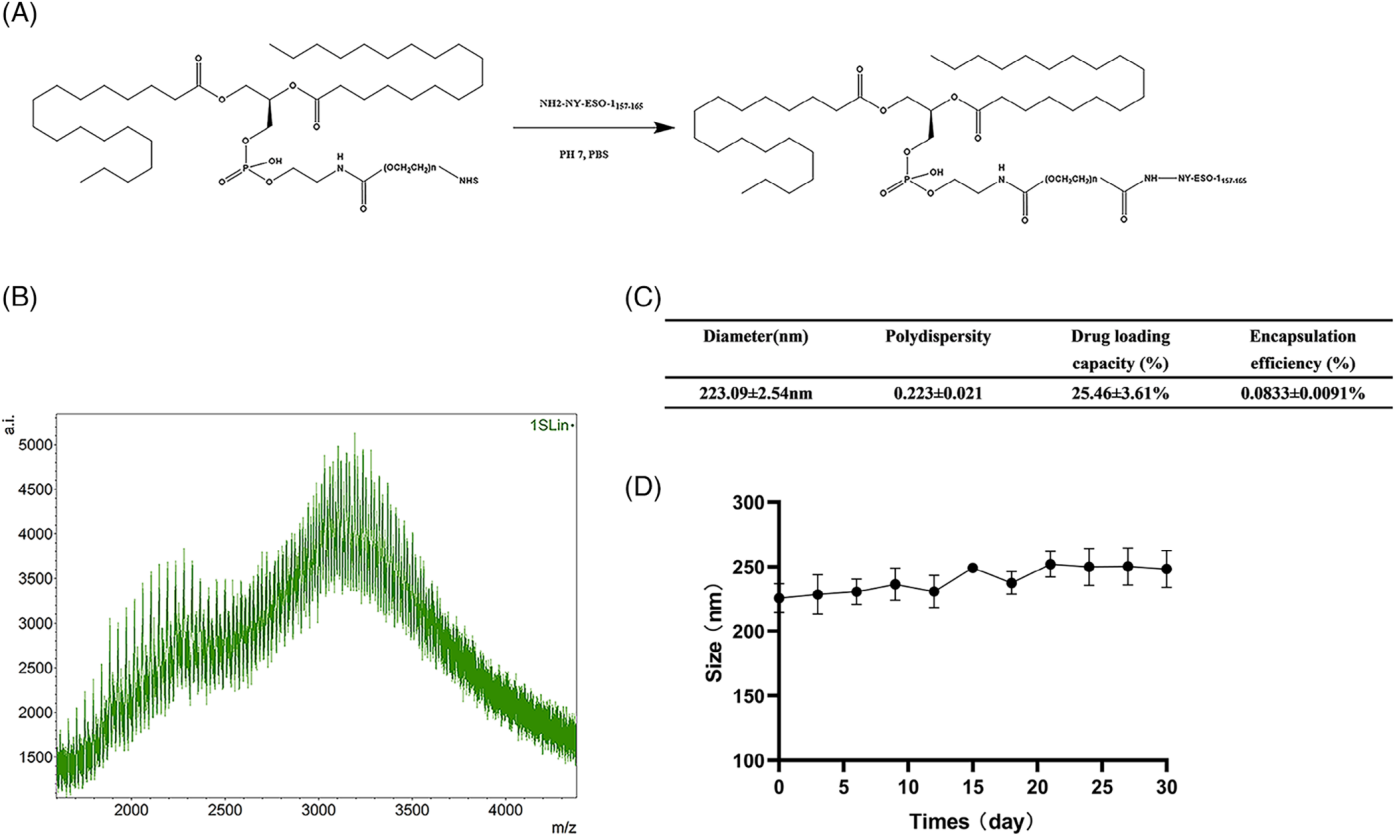
Characterization of NY‐ESO‐1 Lips. (A) A diagram showing the process of creating DSPE‐PEG‐NY‐ESO‐1 conjugate; (B) matrix‐assisted laser desorption/ionization time of flight mass spectrometry (MALDI‐TOF) analysis confirming the linking of NY‐ESO‐1 and DSPE‐PEG‐NHS in the construct; (C and D) evaluation of particle size, drug loading capacity, encapsulation efficiency, and stability of NY‐ESO‐1 liposomes.

The DSPE‐PEG‐NY‐ESO‐1 conjugate was synthesized as a carrier in order to efficiently transport antigens to cancer cells, promoting immunological rejection via neutralization. DSPE‐PEG‐NHS was selected as the backbone of numerous biocompatible polymers due to its distinct structure, excellent biocompatibility, and degradability. The preparation method and characterization of DSPE‐PEG‐NY‐ESO‐1 conjugate are shown in Figure 1A,B. Their average particle size was 223 nm (Figure 1C). The high‐performance liquid chromatography (HPLC) assay determined that 250 μg of NY‐ESO‐1 peptides could be loaded per 3 mg of DSPE‐PEG‐NY‐ESO‐1 conjugate in terms of drug capacity. NY‐ESO‐1 Lips was stable in vitro (Figure 1D).

### 2.2 Safety analysis of the DSPE‐PEG‐NY‐ESO‐1 conjugate

**FIGURE 2:**
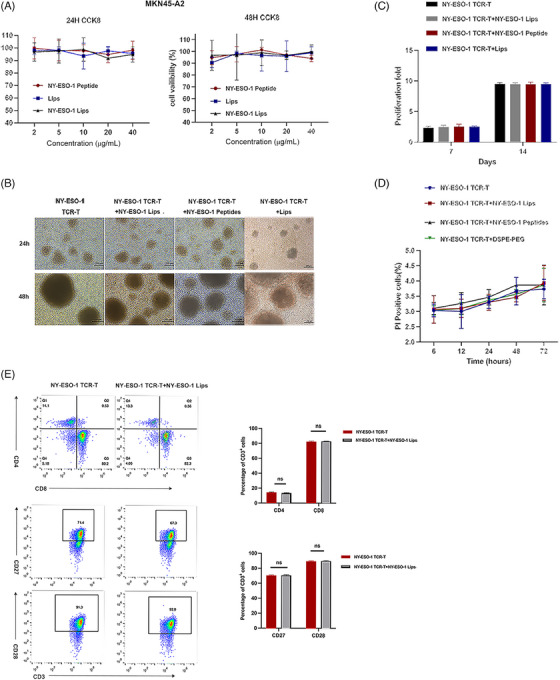
Safety analysis of NY‐ESO‐1 Lips. (A) Cytotoxicity of NY‐ESO‐1 Lips, Lips, and NY‐ESO‐1 peptide on MKN45‐A2 tumor cells was assessed using the CCK‐8 assay; (B) an image of NY‐ESO‐1 TCR‐T cell morphology was captured; (C) the quantity of NY‐ESO‐1 TCR‐T cells was measured on day 7 and 14; (D) the quantity of propidium iodide (PI) positive NY‐ESO‐1 TCR‐T cells was determined using flow cytometry; and (E) we analyzed the phenotypes of cultured lymphocytes using flow cytometry. Data are represented as mean ± SEM; *n* = 3. Data are examined using the Student's *t*‐test unless otherwise noted; significance levels are denoted as *p < 0.05; **p < 0.01; ***p < 0.001. ns, not significant.

We then explored the in vitro toxicity of NY‐ESO‐1 Lips to tumor or T cells. CCK‐8 assay reveled that NY‐ESO‐1 Lips, liposomes (Lips), and NY‐ESO‐1 peptides had little toxicity to tumor cells (Figure 2A). Furthermore, NY‐ESO‐1 TCR‐T cells treated with NY‐ESO‐1 peptides, Lips, or NY‐ESO‐1 Lips exhibited comparable cell morphology, proliferation, viability, and phenotype to unaltered T cells (Figure 2B–E).

### 2.3 NY‐ESO‐1 Lips‐mediated cell membrane antigen modification

**FIGURE 3:**
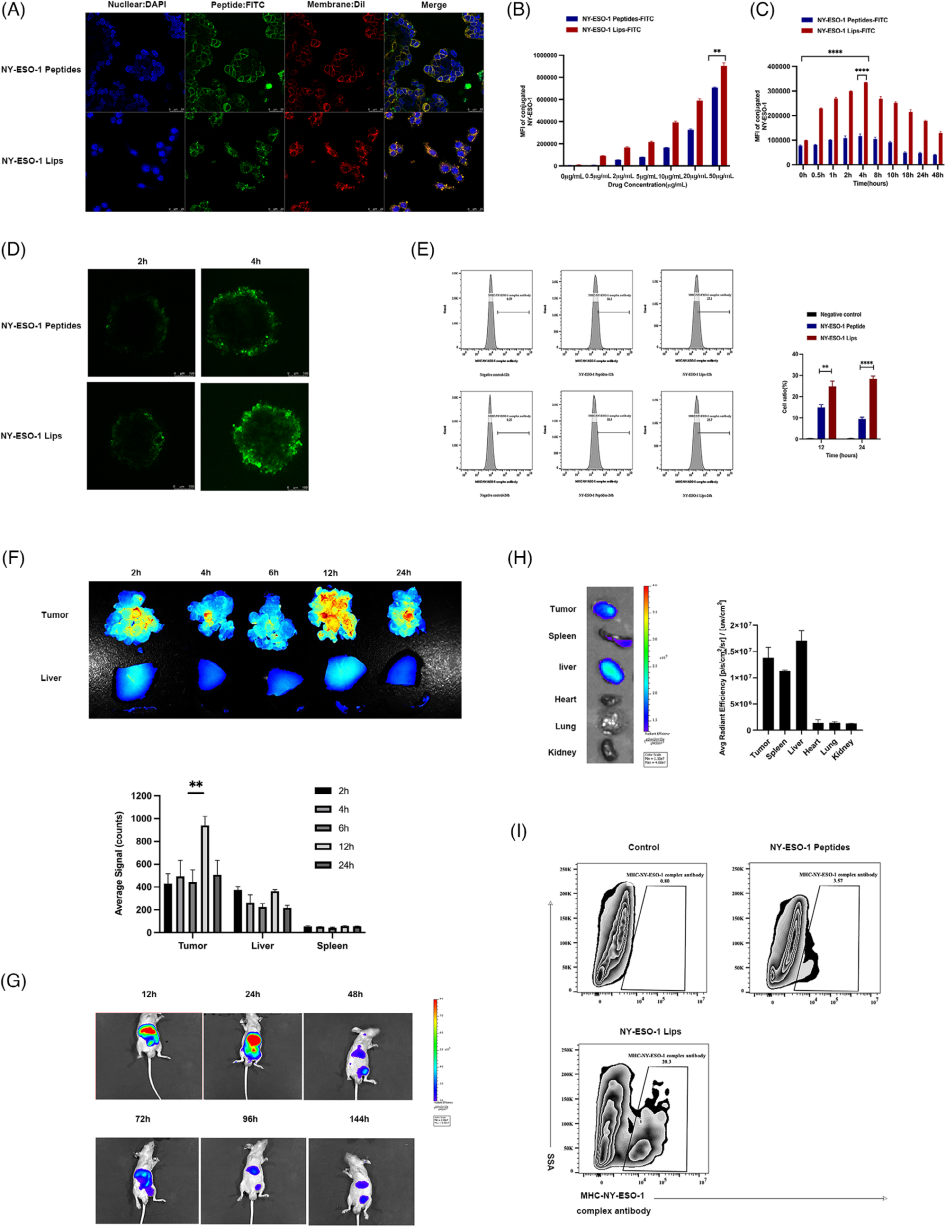
In vitro cellular interaction motif of NY‐ESO‐1 Lips and peptides. (A) Representative confocal images of NUGC4 cells; (B) dose‐dependent binding motif of NY‐ESO‐1 Lips and peptides binding to NUGC4 cancer cells in vitro; (C) effect of incubation time on the levels of NY‐ESO‐1 Lips and peptides binding to NUGC4 cells; (D) Confocal images were taken of HGC27 spheroids to evaluate the ability of FITC‐NY‐ESO‐1 Lips or FITC‐NY‐ESO‐1 peptides to penetrate. Scale bars, 100 μm; and (E) flow cytometry results of MHC‐NY‐ESO‐1 complex expression on MKN45‐A2 tumor cells’ surface. (F) imaging of tumor, liver, and spleen in near‐infrared ex vivo, taken at various time points after injecting DiR‐labeled NY‐ESO‐1 Lips into the NUGC4 peritoneal metastatic model; analyzing the distribution of NY‐ESO‐1 Lips at different time points after injection. Mean fluorescence intensity ± SEM was used to express the results. *n* = 3. Student's *t*‐test results: *p < 0.05; **p < 0.01; ***p < 0.001. (G) In vivo observation of mice with subcutaneous tumors at various time intervals following the injection of NY‐ESO‐1 Lips; (H) visualizations of excised tissues including tumors, livers, spleens, lungs, hearts, and kidneys, captured 144 h after injection (left); semi‐quantitative analysis of NY‐ESO‐1 Lips signal in tumors, livers, spleens, lungs, hearts, and kidneys (right); and (I) flow cytometry assessment of the percentage of tumor cells expressing the MHC‐NY‐ESO‐1 complex.

For cytotoxic T lymphocyte (CTL)‐induced cell death to occur, foreign antigens must first be displayed on target cells' surfaces as a component of the MHC class I complex. Hence, we first investigated interactions between NY‐ESO‐1 Lips and tumor cells. If the NY‐ESO‐1 lips could be absorbed by tumor cells or integrated into the cell membrane or fused with tumor cells, fluorescein isothiocyanate (FITC)‐labeled NY‐ESO‐1 Lips were cocultured with NUGC4 cells, and their interaction motif was observed utilizing confocal microscopy. In Figure 3A, it was observed that the fluorescent peptides were detected on the cell membranes rather than in the cytoplasm, suggesting that NY‐ESO‐1 Lips primarily attach to the tumor cell membrane through direct insertion, followed by the presentation of released peptides on MHC class I molecules of the tumor cells.^21^ Additionally, the NUGC4 cells treated with NY‐ESO‐1 Lips showed a higher fluorescent signal and more FITC‐labeled cells compared to the group treated with NY‐ESO‐1 peptides, indicating a more potent antigen‐modifying ability of NY‐ESO‐1 Lips.

By varying the dose of NY‐ESO‐1 Lips, the density of cell surface antigens could be controlled, suggesting the safety and feasibility of this cell‐surface antigen modification strategy (Figure 3B,C). Furthermore, NY‐ESO‐1 Lips exhibited a higher ability to modify cell‐surface antigens compared to NY‐ESO‐1 peptides. Based on those results, the subsequent in vitro experiment selected 10 μg/mL as the equivalent antigen peptide dose (Figure 3B). When FITC‐labeled NY‐ESO‐1 Lips or NY‐ESO‐1 peptides were co‐cultured with NUGC4 cells for 2 h, the remaining Lips or peptides were removed by washing with Phosphate Buffered Saline (PBS) for three times, and the fluorescence signals existing in tumor cells were evaluated at different time points using flow cytometry. The fluorescence signal of tumor cells gradually increased, peaked at 4 h, and then gradually declined (Figure 3C). Thus, we hypothesized that the NY‐ESO‐1 Lips were effectively inserted into the gastric cancer cells membranes, and 4 h later, the lips were gradually digested or FITC‐labeled peptides were released from the Lips. Characteristic traits of solid tumors encompass irregular angiogenesis and elevated hydrostatic pressure, hindering the effective penetration of therapeutic agents. Therefore, we utilized an in vitro model of multicellular spheroids (MCS) to mimic 3D cell clusters in the body to investigate if the NY‐ESO‐1 Lips could disseminate within the tumor area through their lipid transfer capability. FITC‐labeled NY‐ESO‐1 Lips and peptides were incubated with HGC27 tumor spheroids for 2 h, and then washed and examined at 2 and 8 h, respectively. The migration signal of FITC from the periphery to the center increased significantly in the NY‐ESO‐1 Lips‐treated spheroids compared to that in the NY‐ESO‐1 peptides‐treated spheroids, suggesting that the NY‐ESO‐1 Lips formulation facilitated better penetration through MCS (Figure 3D). The ex vivo experiments initially supported our theory that these liposomes inserted into membranes could efficiently transport antigens to the tumor cell membrane with improved penetration abilities. To visually detect the proportion of successfully modified tumor cells by flow cytometry, we used a fluorescently labeled monoclonal antibody (LY190604, Nanjing Abingen Biotech Inc.), which identifies the SLLMWITQC‐MHC‐I complex rather than the free peptide itself. Compared with NY‐ESO‐1 peptide‐treated group, a higher ratio of positive cells was measured in the NY‐ESO‐1 Lips‐treated group (Figure 3E). The findings indicated that the externally administered NY‐ESO‐1 peptides via NY‐ESO‐1 Lips could effectively be displayed by MHC‐I molecules on cancer cells.

To assess the in vivo distribution of substances, we conducted real‐time near‐infrared fluorescence imaging in a mouse model of gastric cancer with peritoneal spread. In Figure 3F, it is evident that NY‐ESO‐1 Lips labeled with DIR could gather in the metastatic nodules 12 h after celiac infusion, showing a much stronger fluorescence signal in tumor nodules compared to the liver, with statistical significance (*p* < 0.005). We also proved that NY‐ESO‐1 liposomes could home and penetrate in a subcutaneous tumor model. Moreover, in the subcutaneous tumor model, NIR797‐labeled NY‐ESO‐1 Lips were primarily retained in the liver 12 h after systemic administration of NY‐ESO‐1 Lips, with blurred fluorescence signals in the tumor region. Over time, the fluorescence signals of NY‐ESO‐1 Lips were gradually detected in tumor sites, with the strongest signal measured at 24 h, which then declined until 144 h after treatment. After 144 h, the tumors were resected and measured ex vivo. The fluorescence signal present mainly in the tumor nodes and liver (Figure 3G,H). These results implied that the NY‐ESO‐1 Lips efficiently aggregated at the tumor region in vivo and may have the potential to effectively deliver antigens to the tumor.

In order to successfully eliminate tumors with CTLs, tumor cells need to display the MHC‐NY‐ESO‐1 peptide complex in the body. Tail veins injection of NY‐ESO‐1 Lips and NY‐ESO‐1 peptides to tumor bearing mice were performed to evaluate the presentation of NY‐ESO‐1 antigens in vivo. Flow cytometry was used to assay the amount of MHC‐NY‐ESO‐1 complex in the MKN45‐A2 tumor cells 24 h after injection. Significantly, tumor‐bearing mice that received the NY‐ESO‐1 Liposomes (20.3 ± 0.26%) exhibited a 5.41‐fold increase in tumor cells expressing NY‐ESO‐1 antigens compared to those treated with NY‐ESO‐1 peptides (Figure 3I). In addition, the percentage of liver cells displaying the MHC‐NY‐ESO‐1 peptide complex on their surface due to the NY‐ESO‐1 Lips was significantly lower when compared to tumor cells (0.11% vs. 13.7%) as shown in Figure S4. Thus, it was clear that NY‐ESO‐1 had been effectively transported and displayed on MHC class I molecules in the tumor sites by NY‐ESO‐1 Lips.

### 2.4 NY‐ESO‐1 Lips‐mediated antigen modification cooperates with NY‐ESO‐1 TCR‐T‐cell therapy in vitro

**FIGURE 4:**
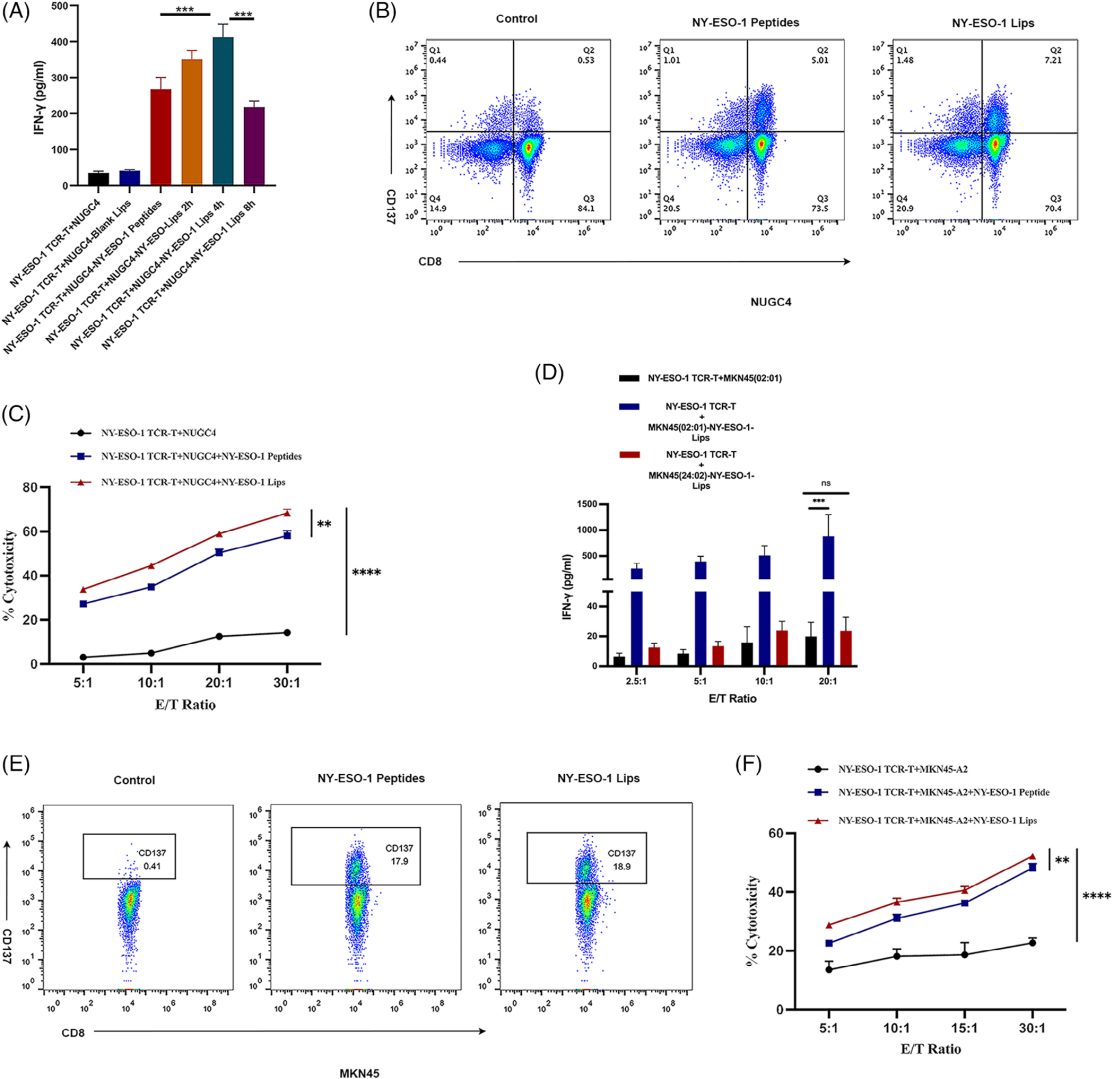
(A and D) Assessment of IFN‐γ level in coculture supernatants (A: NUGC4 tumor cells as target tumor cells; D: MKN45 and MKN45‐A2 tumor cells as target cells); (B and E) flow cytometry results of CD137 expression on NY‐ESO‐1 TCR‐T cells after coculture with specific reagents (B: NUGC4; E: MKN45‐A2); (C and F) monolayer tumor cells were lysed at specified E:T ratios (E: NY‐ESO‐1 TCR‐T cells, T: tumor cells). The findings were presented as the average plus or minus the standard error of the mean. *n* = 3. Student's *t*‐test results: *p < 0.05; **p < 0.01; ***p < 0.001.

We conducted an in vitro experiment to determine if NY‐ESO‐1 TCR‐T cells could identify tumor cells that had been altered with NY‐ESO‐1 Lips. NY‐ESO‐1 TCR‐T cells exhibited recognition of the NY‐ESO‐11_57‐165_ epitope restricted by HLA‐A 02:01. NY‐ESO‐1 TCR‐T cells were activated to secrete IFN‐γ upon binding to NY‐ESO‐1/MHC‐I complexes with their T‐cell receptors. NY‐ESO‐1 TCR‐T cells were generated using an electric transfection method, and the effectiveness of the PiggyBac (PB) and Sleeping Beauty (SB) electric transfection systems was examined (Figure S1). Exposure of NUGC4 tumor cells to NY‐ESO‐1 liposomes resulted in significantly greater activation of NY‐ESO‐1 TCR‐T cells in comparison to treating with unbound peptide at equivalent concentrations (Figure 4A,B). These findings suggest that NY‐ESO‐1 Lips are potentially highly effective against tumors. Furthermore, pretreating NUGC4 cancer cells with NY‐ESO‐1 Liposomes for 4 h led to the strongest stimulation of NY‐ESO‐1 TCR‐T‐cells, consistent with earlier research. MKN45 and MKN45‐A2 cancer cells were utilized to show that HLA‐A 02 01 limits detection by NY‐ESO‐1 TCR‐T cells (Figure 4D,E). Activation of NY‐ESO‐1 TCR‐T cells was induced by NY‐ESO‐1 Lips treatment in MKN45‐A2 tumor cells, while no activation was detected in NY‐ESO‐1 TCR‐T cells treated with NY‐ESO‐1 Lips in MKN45 cells. Furthermore, a significant amount of IFN‐γ was secreted even at a low ratio of effector/targeted cells (E/T), indicating a strong antitumor response by NY‐ESO‐1 TCR‐T.

The tumor cell viability was measured to provide additional clarity on the antigen specificity of the NY‐ESO‐1 Lips‐induced antitumor reaction. The findings from the study showed that NY‐ESO‐1 Lips and NY‐ESO‐1 TCR‐T cells independently demonstrated significant cytotoxicity against gastric tumor cells that did not express the target antigens. Conversely, the coexistence of NY‐ESO‐1 Lips and NY‐ESO‐1 TCR‐T cells resulted in a higher number of deceased cells and a lower number of viable cells (Figure 4C,F). Additionally, the pairing of NY‐ESO‐1 Lips with NY‐ESO‐1 TCR‐T exhibited increased cellular cytotoxicity in comparison to the NY‐ESO‐1 peptides cohort, indicating a superior antigen alteration capability of NY‐ESO‐1 Lips (**p = 0.0017 and **p = 0.0020, respectively). The above ex vivo experiments initially verified our speculation that antigen modification mediated by NY‐ESO‐1 Lips could effectively activate the corresponding TCR‐T cells and work together to exert an antitumor response.

### 2.5 NY‐ESO‐1 Lips facilitate in situ antigen modification to bolster the effectiveness of TCR‐T cells adoptive transfer, a universal therapy for solid tumors

**FIGURE 5:**
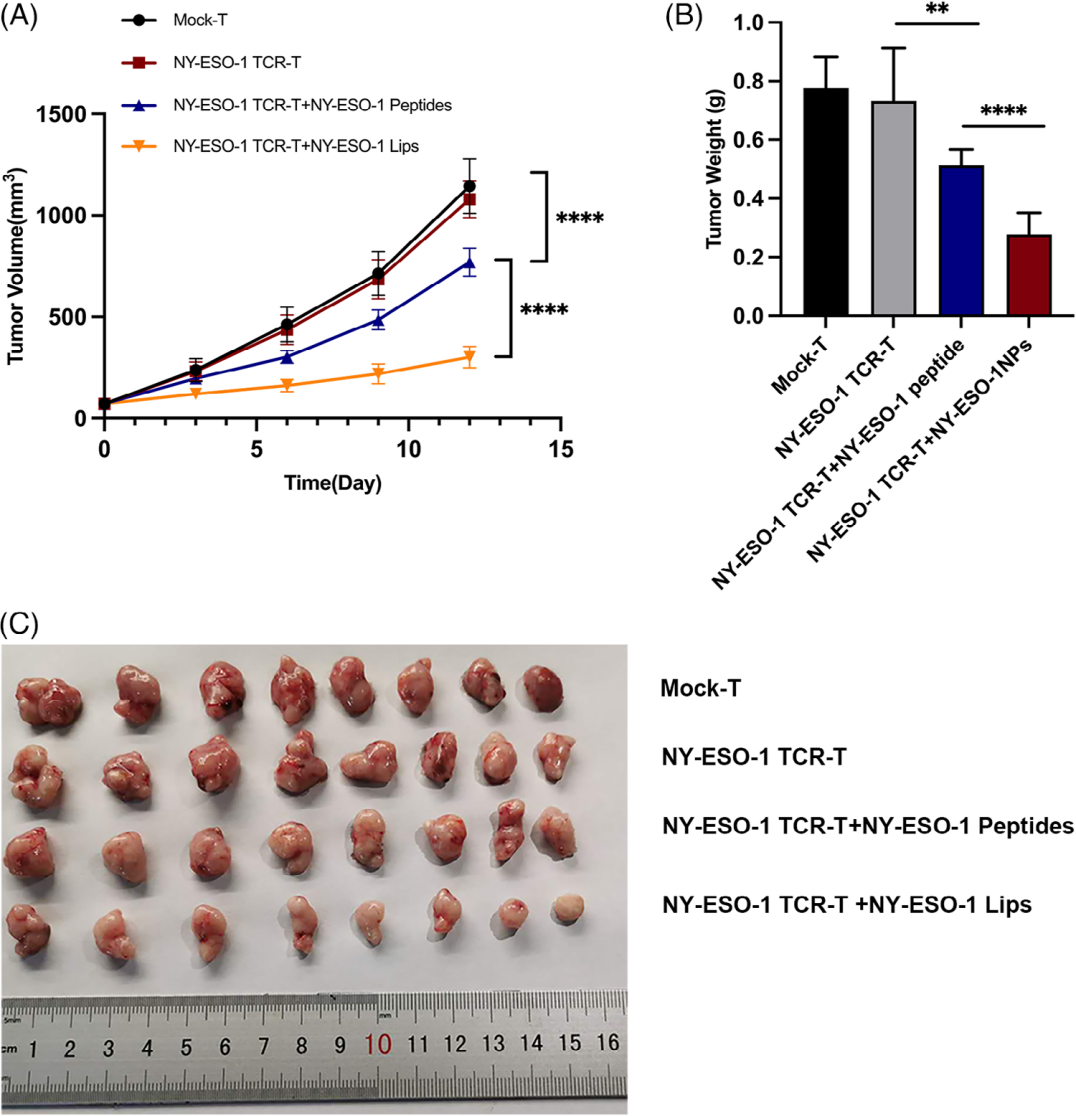
NY‐ESO‐1 TCR‐T cells combined with NY‐ESO‐1 Lips successfully suppressed tumor development. (A) Mean tumor volume was used to represent tumor growth, with error bars indicating standard deviation (SD); ****p < 0.0001 compared with the treatment group (Mann–Whitney *U* test). Eight mice were used in each group. (B) Tumor masses in each group were measured and (C) photographed with a sample size of 8. Unless otherwise stated, data are analyzed using the Student's *t*‐test and presented as mean ± SEM. Statistical significance is denoted as *p < 0.05; **p < 0.01; ***p < 0.001. ns, not significant.

In vitro studies have proved the success of tumor cell antigen modification by NY‐ESO‐1 Lips, and we further studied antitumor efficacy of this combination therapy in vivo. We generated an MKN45‐A2 subcutaneous tumor‐bearing model. Initially, we demonstrated that the NY‐ESO‐1 peptides in conjunction with NY‐ESO‐1 TCR‐T‐cell therapy are effective in suppressing tumor progression when compared to NY‐ESO‐1 TCR‐T treatment alone (****p < 0.0001) (Figure 5). Furthermore, the combination of NY‐ESO‐1 Lips and NY‐ESO‐1 TCR‐T ‐cell therapy demonstrated notably superior antitumor effectiveness compared to NY‐ESO‐1 peptides in conjunction with NY‐ESO‐1 TCR‐T cells (****p < 0.0001).

**FIGURE 6:**
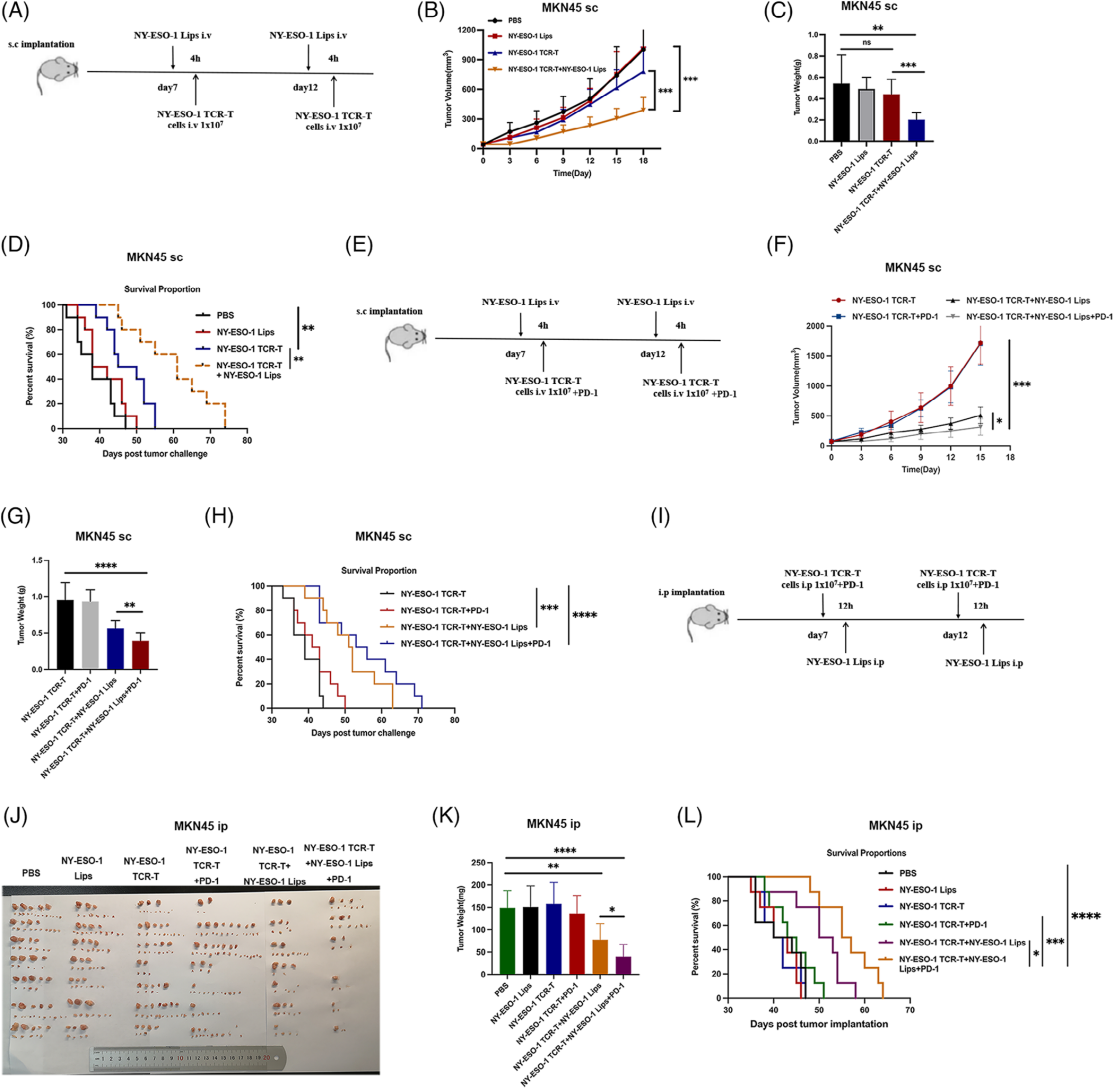
The combination of NY‐ESO‐1 TCR‐T cells with NY‐ESO‐1 Lips and PD‐1 blockade successfully suppressed tumor progression. (A and E) Illustration showing the treatment process in a subcutaneous tumor model. (B–H) Tumor growth was quantified using the mean tumor volume, represented by bars indicating standard deviation (SD). Statistical significance was observed with a ***p value < 0.001 when comparing the treatment group to the control groups using the Mann–Whitney *U* test. Kaplan–Meier survival curves demonstrated significantly improved survival in the treatment group compared to the control groups (**p < 0.01, log‐rank test). Each experimental group comprised eight mice. (I) Diagram showing the treatment procedure for MKN45‐A2 peritoneal metastasis tumor model, *n* = 7. On day 11 following tumor implantation (day 11), the administration of either 1 × 10^7^ NY‐ESO‐1 Lips alone or in combination with NY‐ESO‐1 TCR‐T cells, or NY‐ESO‐1 TCR‐T cells alone, was performed intraperitoneally every 3 days, resulting in a total of two doses being given. Intraperitoneal administration of 250 μg of PD‐1 inhibitor was done every 3 days for a total of two doses. Tumors were harvested after 20 days of treatment and weighed. (J) Tumor nodules in each experiment group were pictured and (K) subsequently weighed in the MKN45‐A2 model of peritoneal metastatic tumors. (L) Survival analysis in MKN45‐A2 peritoneal metastatic tumor model treated with PD‐1, *n* = 8. Data are analyzed with Student's *t*‐test unless otherwise stated and are represented as mean ± SEM; *p < 0.05; **p < 0.01; ***p < 0.001. ns, not significant.

In the model of MKN45‐A2 subcutaneous tumors, the combined therapy of NY‐ESO‐1 TCR‐T cells and NY‐ESO‐1 Lips had a synergistic effect on suppressing tumor growth and extending survival time to some degree, while NY‐ESO‐1 Lips by itself had minimal impact on inhibiting tumor growth (Figure 6B–D). As expected, maximum antitumor efficacy was evoked in both tumor controls when combined with the PD‐1 blockage. In addition, the survival rate was prolonged (Figure 6E–H). After 50 days, 60% of the mice in the NY‐ESO‐1 TCR‐T + NY‐ESO‐1 Lips + PD‐1 and NY‐ESO‐1 TCR‐T + NY‐ESO‐1 Lips group remained alive, while all mice in the other control groups had died. Moreover, a survival rate of 30% was recorded among the mice in the NY‐ESO‐1 TCR‐T + NY‐ESO‐1 Lips + PD‐1 treatment group at the end of 63 days, while all mice in the NY‐ESO‐1 TCR‐T + NY‐ESO‐1 Lips group succumbed to mortality. The MKN45‐A2 peritoneal metastatic tumor model was established to evaluate antitumor efficacy of NY‐ESO‐1 Lips in vivo (Figure 6I). Mice that received a combination of NY‐ESO‐1 TCR‐T and NY‐ESO‐1 Lips showed significantly reduced tumor sizes and increased survival time compared to those treated with PBS, NY‐ESO‐Lips, or NY‐ESO‐1 TCR‐T by itself (Figure 6J–L). PD‐1 blockage could further suppress tumor growth and extend survival significantly. In addition, we observed a superior ability to inhibit liver metastasis in the combination treatment group (Figure S3D). Further investigation was conducted on the benefits of combination treatment in the NUGC4 peritoneal metastatic mouse model, showing comparable anti‐cancer outcomes (Figure S3A–C). None of the experimental groups exhibited any changes in body weight or in the histological staining of vital organs such as lungs, liver, heart, kidneys, and spleen using hematoxylin and eosin (H&E) staining (Figure S2). The evidence presented showed the positive safety characteristics of NY‐ESO‐1 Lips and NY‐ESO‐1 TCR‐T.

In summary, the findings demonstrate that NY‐ESO‐1 Lips effectively alter malignant cells with NY‐ESO‐1 peptides, which are targeted and destroyed by NY‐ESO‐1 TCR‐T cells. The combination of PD‐1 blockade produced profound antitumor effects.

## 3 DISCUSSION

Our team has been committed to the universalization of NPs for a long time. Sun and his team were the first to develop a universal CAR‐T‐cell therapy that targets antigens, using a nanosystem that fuses with cells.^22^ In Wang's research, gelatinase‐responsive NPs were used to create a “NanoSwitch” that can activate CAR‐T cells at the tumor site, addressing safety issues with CAR‐T therapy regardless of the target.^23^ In this study, a “universal” TCR‐T‐cell therapy was developed by modifying tumor cell antigens externally. The universal TCR‐T therapy can enable tumor cells to be identified and killed by TCR‐T cells regardless of antigen expression, tumor HLA typing, or TCR‐T type. This strategy is universal to antigens, which means any immunogenic peptides can be modified to tumors cell though this strategy and provide targets for specific TCR‐T cells. Besides, this strategy is also universal as to HLA typing. Tumor cells with different HLA typing can be artificially modified with relative HLA‐restricted peptides and be recognized by specific TCR‐T cells, since there are several TCR‐T products (such as MAGE‐A4 TCR‐T and MSLN TCR‐T) that have entered clinical trial. The versatility of this approach extends to TCR‐T cells, as tumors can be engineered to present various peptides that can be detected by matching TCR‐T cells, expanding their potential use to a wider range of patients. It has been widely studied that extracellular vesicle showed a potential of tumor cells to modify antigens preferentially in tumor microenvironment.^24^ Vesicles naturally form through processes such as secretion (exocytosis), uptake (phagocytosis and endocytosis), and material transport within the cytoplasm. Moreover, they can be artificially manufactured, which is so‐called liposomes.^25^ In our study, we leveraged the membrane insertion and fusion ability of liposomes^20^, ^26^ to distribute NY‐ESO‐1 antigen on the surface of gastric tumor cells in the form of peptide–MHC complexes. Moreover, the membrane fusion NPs have the ability to mediate intercellular lipid transfer,^27^ which showed more tumor infiltration capacity. The mechanism of antigen modification process by liposome was unclarified. According to previous studies,^20^ we hypothesized that antigen‐loaded liposomes inserted and fused to tumor membrane through the exchanging process of lipids at membrane contact sites. The versatility of this approach extends to TCR‐T cells, as tumors can be engineered to present various peptides that can be detected by matching TCR‐T cells, expanding their potential use to a wider range of patients.^21^ MKN45‐A2 and NUGC4 cells were selected as ALVs because they lack endogenous expression of the NY‐ESO‐1157‐165 epitope, making them unable to be targeted and attacked by NY‐ESO‐1 TCR‐T cells. Both in laboratory and living organisms research demonstrated the presence of NY‐ESO‐1 MHC‐I complex on the surface of tumors, supporting the previous assumption, whereas detailed explements were needed to further elucidate the process of antigen modification mechanism.

The incidence of gastric cancer ranks fifth and the third most lethal,^28^ with a high likelihood of primary tumor growth, peritoneal spread, and liver and distant LN metastases.^29^ To address clinical demands, we developed models of peritoneal metastasis tumors and subcutaneous tumors to investigate the potential use of universal TCR‐T therapy in treating gastric cancer. Liposome‐based antigen modification strategy could improve gastric cancer immunogenicity, resulting in a greater antitumor effect. We delivered liposome antigen peptide through intraperitoneal administration, which significantly reduced the number and size of peritoneal metastatic nodules. NY‐ESO‐1 TCR‐T cells + Ag‐NPs combination therapy effectively suppressed liver metastatic tumor nodules in peritoneal metastasis tumor models, with no observed autoimmune reaction in liver tissue. The reason for this may be that between 30% and 99% of NPs given are likely to gather in the liver after being introduced into the body, as shown in near‐infrared fluorescence imaging.^30^ Hence, the liver accumulation feature could be exploited for treating liver metastasis nodes, potentially expanding the clinical use of NY‐ESO‐1 TCR‐T therapy in liver metastasis tumors.

In traditional cancer therapy, liposomes, as chemotherapy drug carriers, have active tumor targeting ability and they uptake via receptor‐mediated endocytosis.^31^ However, this targeting ability is not specific enough, resulting in accumulation in normal tissues^32^ and bringing corresponding drug toxicity. In addition, the dense obstacles present at the tumor location could hinder the liposomes' ability to find and enter tumors, potentially leading to unintended toxicity.^33^ Our research team has previously demonstrated a specific tumor membrane preference for the modified antigen pattern, while the delivery of antigens to normal cells' membranes was ineffective.^22^ Moreover, past studies have shown that although normal cells can take up NPs, their expression of certain antigen–MHC complexes is low, resulting in insufficient immune response for TCR‐T cells due to low antigen levels and cellular protection mechanisms.^34^ As a result, our study developed a liposome‐based antigen carrier specifically targeting tumors with enhanced safety measures.

A limitation of this strategy is its delivery method. As we can see, the delivery system was not specific to tumor region. To further optimize the tumor‐specificity of NPs, tumor response or tumor cell targeting elements could be introduced, such as IRGD peptides or gelatinase cleavable peptide that reacts to tumor microenvironment as a result of multiple enzymes overexpressed.^35^, ^36^ Another constraint is its dependence on the existence of MHC class I molecules. The theoretical failure of this approach could occur if tumors experience a decline in MHC class I expression. This obstacle can be addressed by upregulating the expression of MHC I molecules on tumor cells or directly modifying MHC‐peptide complexes onto tumor cells surface.

**FIGURE 7:**
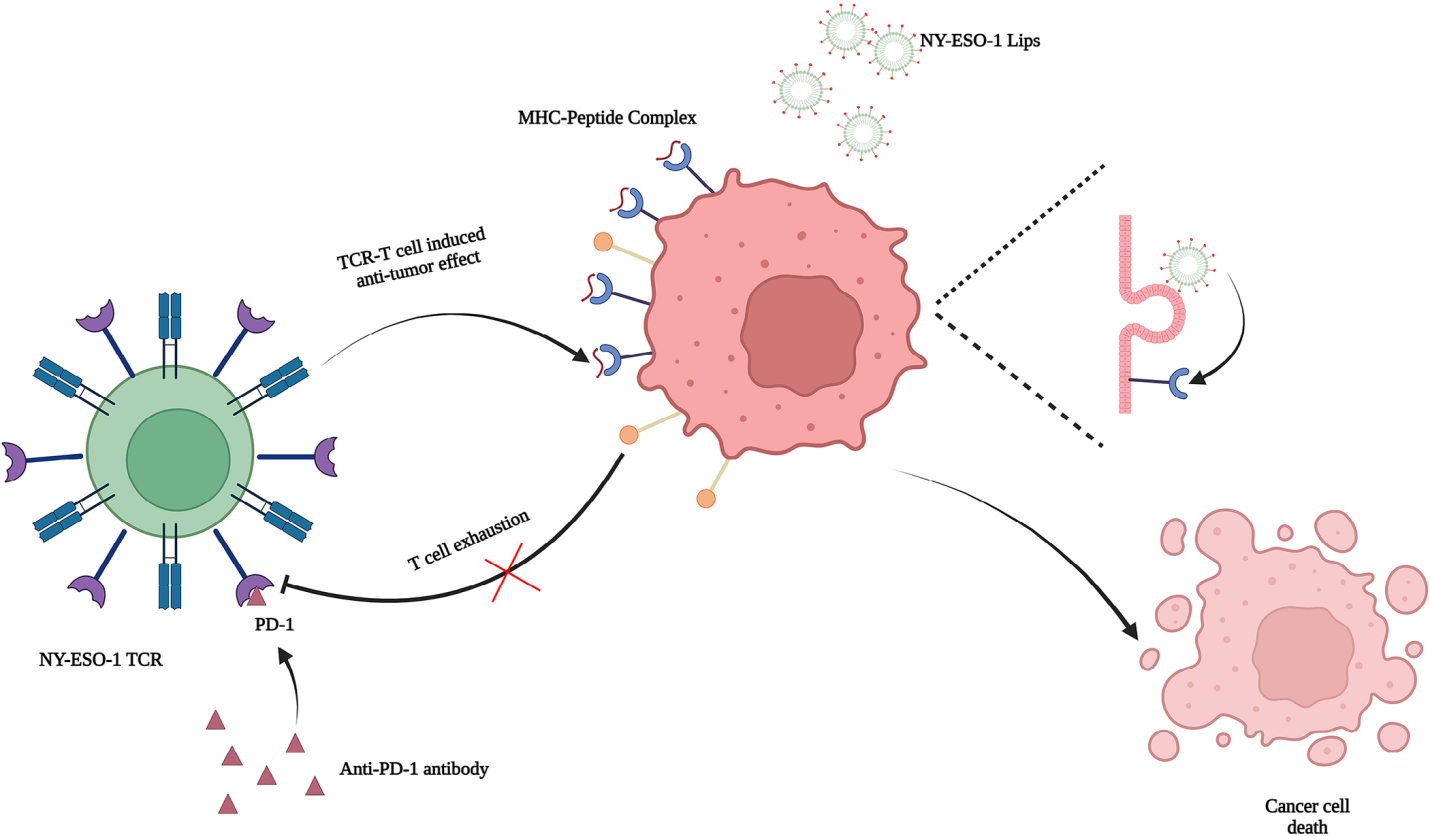
NY‐ESO‐1 Lips delivers the antigen to tumor cells in order to “arm” the tumor. NY‐ESO‐1 TCR‐T cells target the antigen, triggering an anti‐cancer response that leads to the destruction of cancer cells. Additionally, anti‐PD‐1 antibody can enhance the therapeutic effect.

Altogether, our research provided a novel strategy for broaden application of TCR‐T therapy, which was not restricted to peptide delivery system or TCR‐T itself. Our study mainly focused on solving the different expressions of certation antigen between tumor cells in same tumor or different tumors by cell membrane antigen modification strategy. In addition, administering NY‐ESO‐1 peptides using NY‐ESO‐1 Lips to two ALVs solid tumor models resulted in a considerable reduction in tumor growth and improved tumor elimination during TCR‐T therapy. Ultimately, the in situ alteration of antigens has greatly enhanced the safety and accessibility of TCR‐T therapy, offering fresh insights for the future implementation of solid tumor treatment in clinical settings. Besides, by delivering different modification elements, these cooperating therapies can be used for neither innate nor adoptive immune therapy, which provides new perspectives for solid tumor immunotherapy (Figure 7).

## 4 METHODS

### 4.1 Preparation and characterization of NY‐ESO‐1 Lips

The DSPE‐PEG‐NY‐ESO‐1 was synthesized by conjugating 1,2‐distearoyl‐sn‐glycero‐3‐phosphoethanolamine‐N‐[NHS‐(polyethylene‐glycol)−2000] (DSPE‐PEG_2000_‐NHS) (Laysan Bio, Inc.) with NY‐ESO‐1_157‐165_ peptide (Top‐Peptide). Briefly, DSPE‐PEG‐NHS was quickly combined with NY‐ESO‐1_157‐165_ or NY‐ESO‐1_157‐165_‐FITC in a 1:1 molar ratio in Hepes buffer (pH 7.4). After stirring the mixtures with a magnetic stirrer at room temperature for 24 h, the crude product was moved to dialysis tubing with a 3000 molecular mass cut off. It was then dialyzed in deionized water for another 24 h to eliminate any remaining NY‐ESO‐1_157‐165_. The DSPE‐PEG‐NY‐ESO‐1 product was achieved through freeze‐drying, and subsequently kept at −20°C. Product identity was verified with MALDI‐TOF‐MS (Bruker Daltonics). The DSPE‐PEG‐NY‐ESO‐1 yield was assessed using HPLC analysis (BestBio). The NY‐ESO‐1 Lips were analyzed for their average diameter and distribution of sizes using dynamic light scattering (DLS) with Brookhaven BI‐9000AT (Brookhaven Instruments Corporation). Zeta potential analysis was performed using Zeta Plus, a laser Doppler anemometer (Brookhaven Instruments Corporation). At least three repetitions were conducted for each measurement. NY‐ESO‐1 Lips were stored at ambient temperature. DLS was used to measure the dimensions of NY‐ESO‐1 Lips every other day over a period of 15 days in order to evaluate their consistency.

### 4.2 Cell culture

Prior to experimentation, the human gastric adenocarcinoma cell lines MKN45 and NUGC4 were acquired from the Cell Bank of Shanghai Institute of Biochemistry and Cell Biology. They were then grown in RPMI 1640 medium supplemented with 10% fetal calf serum, penicillin at a concentration of 100 U mL^−1^, and streptomycin at a concentration of 100 μg mL^−1^. MKN45‐A2 (a gastric cancer cell line transfected with HLA‐A02:01molecular) was transferred by iCarTab Biotechnology Co. Ltd. All the cells were maintained at a temperature of 37°C and an atmosphere of 5% CO_2_ during incubation, and their authenticity was confirmed by examining their morphology under a microscope after being plated at various concentrations. Only cells that were free of Mycoplasma were utilized after being tested for the presence of the bacteria.

### 4.3 Generation of NY‐ESO‐1 TCR‐T cells

The blood collection procedure was meticulously carried out, adhering strictly to the sanctioned guidelines issued by the Ethics Committee at Nanjing Drum Tower Hospital. Every contributor provided a signed informed consent form for authorizing participation in the scientific research. Peripheral blood mononuclear cells (PBMCs) were obtained from healthy volunteer samples using centrifugation on a Ficoll density gradient and then resuspended in AIM‐V medium (Gibico). PBMCs were incubated by sticking for a duration of 2 h, while T lymphocytes that did not stick were grown in a complete medium consisting of 90% AIM‐V (Gibico) and 10% fetal bovine serum (FBS) (Gibico), and stimulated with 50 ng/mL OKT3 (eBioscience) and 300 U/mL IL‐2 (Peprotech) for a period of 2−3 days. NY‐ESO‐1 TCR‐T cells were generated by introducing the designated plasmids into OKT‐3‐activated T cells via Nucleofector 2B (Lonza). Note that 10^7^ cells were washed with PBS and resuspended in 100 μL transfection buffer (Amaxa Human T cells Nucleofector Kit, VPA‐1002; Lonza). The transfection program was Program T‐007. Following transfection, cells were resuspended in 500 μL per‐warmed AIM‐V medium, and every 2−3 days, half of the culture medium was replaced with fresh complete medium containing 100 U/mL of IL‐2.

### 4.4 Cell viability assay

The MKN45‐A2 cells were digested, collected, and seeded into 96‐well plates with a density of 3 × 10^3^ cells per well. The MKN45‐A2 cells were exposed to PBS, NY‐ESO‐1 Lips (at concentrations equivalent to 2, 5, 10, 20, and 40 μg/mL peptides), as well as Lips and NY‐ESO‐1 peptides (at concentrations of 2, 5, 10, 20, and 40 μg/mL) on the second day. Note that 10 μL of CCK‐8 Solution (Vazyme Biotech Company) was added after 24 and 48 h. The microplate reader (TECAN) was used to measure the absorbance at 450 nm for each well. In the TCR‐T‐cell proliferation test, TCR‐T cells were grown either by themselves or with NY‐ESO‐1 Lips, NY‐ESO‐1 peptides, Lips, and then expanded in a full complete medium consisting of 90% AIM‐V (Gibico), 10% FBS (Gibico), and 100 U/mL IL‐2 (Peprotech). Next, images of NY‐ESO‐1 TCR‐T cell morphology images were captured, and the quantities of NY‐ESO‐1 TCR‐T cells were tallied on both the 7th and 14th days. Flow cytometry was used to measure the percentage of NY‐ESO‐1 TCR‐T cells that were propidium iodide (PI) positive at various time points (6, 12, 24, 48, and 72 h) to assess the safety of NY‐ESO‐1 liposomes, NY‐ESO‐1 peptides, and liposomes on NY‐ESO‐1 TCR‐T cells. NY‐ESO‐1 TCR‐T cells were analyzed for phenotype expression using flow cytometry following treatment with PBS, NY‐ESO‐1 Lips, and NY‐ESO‐1 peptides. Flow cytometry was used to analyze anti‐ CD3‐PerCP‐Cy5.5 (MHCD0312; BD Bioscience), anti‐CD4‐FITC (RPA‐T4, BD Bioscience), anti‐CD8‐ APC (RPA‐T8, BD Bioscience), anti‐CD27‐PE (M‐T271, BD Bioscience), and anti‐CD28‐PE (CD28.2, BD Bioscience) were performed.

### 4.5 Cellular insertion behavior of NY‐ESO‐1 lips

To study the cellular insertion ability of the NY‐ESO‐1 Lips in a laboratory setting, the NY‐ESO‐1 Lips were tagged with FITC. NUGC4 cells were grown in RPMI 1640 medium with 10% (v/v) FBS and 1% (v/v) penicillin and streptomycin solution in a humidified cell culture incubator at 37°C. When the cells grew to the logarithmic proliferation stage, they were planted in a 4‐Chamber Glass Bottom Dish (Cellvis). Once the cells had attached to the surface, the culture medium was removed, NY‐ESO‐1 Lips were introduced, and the mixture was left to incubate at 37°C with 5% CO_2_ for 1 h. Afterward, the cells were treated with 4% paraformaldehyde for a duration of 10 min. Following PBS wash, cells were exposed to 0.2% Triton X‐100 and then blocked for 2 h in a 1% bovine serum albumin solution. Cells were then treated with 300 nM DAPI and DiI in PBS, respectively. The conjugate was examined and measured for cellular uptake using confocal microscopy (Carl Zeiss) and flow cytometry (BD Bioscience), respectively.

After seeding cells at a density of 2 × 10^5^ cells per well in 6‐well plates for 1 day, they were exposed to FITC‐labeled NY‐ESO‐1 peptides or NY‐ESO‐1 Lips (NY‐ESO‐1_157‐165_ peptides at doses of 0.5, 2.5, 10, 20, and 50 μg/mL) in RPMI 1640 media without FBS for 2 h. Following this, the cells were rinsed twice with PBS containing Ca^2+^ and Mg^2+^, and analyzed using flow cytometry (BD Air). To detect the duration time of antigen insertion, NUGC4 cells were seeded at a density of 2 × 10^5^ cells/well in six‐well plates for 1 day and treated with FITC‐labeled NY‐ESO‐1 peptides or NY‐ESO‐1 Lips (NY‐ESO‐1_157‐165_ peptides dose 10 μg/mL) in RPMI 1640 media without FBS for 2 h to determine the duration of antigen insertion. After treatment, the cells were then washed twice with PBS containing Ca^2+^ and Mg^2+^ and then cultured in RPMI 1640 media with 10% FBS. The cells were washed and quantified using flow cytometry (BD Air) at various time intervals (0.5, 1, 2, 4, 8, 12, 24, and 48 h).

### 4.6 Experiments in MCSs

The HGC27 cells (500 in 150 μL of complete media) were plated in a 96‐well clear round bottom ultra low attachment microplate (Corning) and incubated at a temperature of 37°C for 72 h until reaching a diameter of approximately 200 μm. Microscopic examination was used to monitor MCSs, and only tumor spheroids that exhibit a uniform and compact morphology were selected for further investigation. FITC‐NY‐ESO‐1 Lips or FITC‐NY‐ESO‐1 peptides were exposed to pre‐existing spheroids for 2 h at 37°C to investigate the penetration of MCSs, followed by washing to eliminate any unbound lips. The spheroids were incubated for an additional 2–8 h to enable the cargo to penetrate further. Tumor spheroids were scanned with a confocal microscope (Leica) after being washed and fixed in 4% paraformaldehyde. Images were captured of the cross‐sections located near the center of the uniformly sized spheroids at specific time intervals using a confocal microscope with 10 μm spacing. The images were subsequently subjected to detailed analysis using Image J software for further investigation.

### 4.7 Detection of NY‐ESO‐1_157‐165_ peptide presentation on MHC‐I of tumor cells by fluorescence‐activated cell sorting (FACS)

MKN45‐A2 cells were treated with increasing concentrations of either free NY‐ESO‐1 peptides or NY‐ESO‐1 Lips and incubated at 37°C in a 5% CO_2_/air incubator for 2 h. Control MKN45‐A2 cells without NY‐ESO‐1 peptide as control group were also incubated under the same conditions. After washing the cells with FACS buffer (1% FBS, 0.5% NaN_3_ in PBS), they were stained with MARFc‐A2/ESO1 (Nanjing Abingen Boptech Inc) bound to SLLMWITQC for 1 h at room temperature. Subsequently, the cells were rinsed once more using FACS buffer and stained with Goat F(ab')2 Anti‐Human IgG—(Fab')2 (DyLight 488) (Abcam) for 30 min at room temperature. After another round of washing with FACS buffer, the cells underwent FACS analysis. The control group exhibited minimal cellular staining compared to the background.

### 4.8 NY‐ESO‐1 TCR‐T cell activation assay

Note that 1 × 10^4^ NUGC4 cells or MKN45 and MKN45‐A2 cells were placed in a 96‐well plate overnight and then treated with either NY‐ESO‐1 peptide or NY‐ESO‐1 Lips (equivalent to 10 μg/mL peptide). Two hours later, the cells were rinsed, resuspended in 100 μL fresh media, and incubated for different time periods (2, 4, and 8 h). Next, 1 × 10^5^ NY‐ESO‐1 TCR‐T cells were introduced and incubated together for another 16 h. The medium was then gathered and the release of IFN‐γ due to NY‐ESO‐1 TCR‐T stimulation was measured using the Human IFN‐γ Flex Set (Bead B8) (BD Biosciences). Negative controls included responses triggered by either no‐peptide or Blank Lips.^37^ The average results were displayed in quadruplicate. Furthermore, flow cytometry was utilized to evaluate the T‐cell activation indicator 4‐1BB (CD137). The antibodies utilized for the flow cytometry analysis were as follows: CD8‐Percp‐Cy5.5 (RPA‐T8, BD Bioscience) and CD137‐APC (4‐1BB, BD Bioscience). Each sample was suspended in FACS buffer, followed by staining with the specified antibodies for 30 min in a dark, 4°C environment. After that, they were washed twice and resuspended in FACS buffer prior to analysis.

### 4.9 Cytotoxicity assay

Transduced T cells were subjected to a cytotoxicity assay using carboxy fluorescein succinimidyl ester (CFSE)/PI labeling.^38^ Before the test, NUGC4 or MKN45‐A2 cells were treated with either free NY‐ESO‐1 peptides or NY‐ESO‐1 Lips (equivalent to 10 μg/mL peptides). Following a 2‐h incubation, the cells were washed, resuspended in 1 mL PBS and labeled with 4 μM CFSE (Invitrogen) for 10 min at 37°C in PBS. The labeling process was halted by introducing a 10‐fold volume of PBS and thoroughly rinsing with PBS before placing in the 24‐well plates. NUGC4 or MKN45‐A2 cells labeled with CFSE were placed in RPMI with 0.5% FBS and then exposed to NY‐ESO‐1 TCR‐T cells at ratios of 5:1, 10:1, 20:1, and 40:1 ratio for 6 h at 37°C and 5% CO_2_. To calculate the proportion of cell death, PI (Sigma) was included. Flow cytometry was used to analyze the samples.

### 4.10 In vivo real time near‐infrared fluorescence imaging

NY‐ESO‐1 Lips were quantitatively localized using in vivo real time near‐infrared fluorescence imaging. The conjugates were tagged with the near‐infrared fluorescent probe, DIR (Bridgen) or NIR797 (Sigma–Aldrich Co.). When NUGC4 or MKN45‐A2 tumor‐bearing mice had tumor volumes reached 150−200 mm^3^, 100 μL of physiological saline containing DIR‐labeled NY‐ESO‐1 Lips or NIR 797‐labeled Lips were injected intraperitoneally or intravenously, respectively (*n* = 3 per group). The Lips were observed at scheduled intervals using a Maestro EX system (Cambridge Research & Instrumentation). To image resected tissue, the mice were euthanized while under deep anesthesia. Tumors and main organs, including heart, liver, spleen, lung, and kidney, were excised and imaged.

### 4.11 In vivo antigen presentation quantification

Quantifying antigen presentation in living organisms involved creating a tumor‐bearing animal model by injecting MKN45‐A2 cells (2 × 10^6^ cells per each mouse) subcutaneously into male nude mice (4‐ to 5‐week old, male). Ten days later, both NY‐ESO‐1 peptides and NY‐ESO‐1 Lips were dissolved in saline with 50 μg of NY‐ESO‐1_157‐165_ and administered intravenously. After 24 h of injection, the tumor tissue was extracted and broken down using a cell strainer and PBS. After staining with MARFc‐A2/ESO1, cells were incubated with SLLMWITQC for 1 h at room temperature in FACS buffer, followed by three washes with FACS buffer. Subsequently, the cells were stained with Goat F(ab')2 Anti‐Human IgG—(Fab')2 (DyLight 488) for 30 min at room temperature in FACS buffer, followed by three more washes with FACS buffer before FACS analysis. Flow cytometry analysis was conducted to quantify the in vivo presentation of NY‐ESO‐1.

### 4.12 Xenograft mouse models

In accordance with the guidelines established by the Animal Care Committee at Drum Tower Hospital, all experiments involving xenograft mouse models were conducted. All experiments in this study were approved by the ethics committee at Drum Tower Hospital. All animal experiments were conducted in accordance with the regulations. The animal studies were not conducted with investigators being unaware of the details. Every attempt was made to reduce the quantity of animals utilized and alleviate their pain. Male BALB/c nude mice, aged 4−5 weeks, were injected with 10^6^ NUGC4 or MKN45‐A2 cells intraperitoneally to create peritoneal metastasis tumor models. Male BALB/c nude mice, aged 4−5 weeks, were injected with 10^6^ MKN45‐A2 cells to create a subcutaneous tumor model.

### 4.13 In vivo antitumor efficacy and safety evaluation

Assessment of the effectiveness and safety of anti‐tumor treatments was conducted in vivo using the NUGC4 model, where mice with peritoneal tumors were divided into four randomized groups. After a span of 10 days, NY‐ESO‐1 Lips (equivalent to 50 μg of NY‐ESO‐1 peptides) were administered intraperitoneally every 5 days for a total of two injections, either in combination with 1 × 10^7^ NY‐ESO‐1 TCR‐T cells or 1 × 10^7^ Mock TCR‐T cells or alone. Tumor‐carrying mice in the MKN45‐A2 subcutaneous tumor model were randomly assigned to seven different groups: PBS, NY‐ESO‐1 TCR‐T, NY‐ESO‐1 peptides, NY‐ESO‐1 Lips, NY‐ESO‐1 peptides + NY‐ESO‐1 TCR‐T, NY‐ESO‐1 Lips + NY‐ESO‐1 TCR‐T, and NY‐ESO‐1 Lips + NY‐ESO‐1 TCR‐T. Tumor‐bearing mice in the MKN45‐A2 peritoneal tumor model were randomly assigned to six different groups. After a span of 10 days, 1 × 10^7^ NY‐ESO‐1 TCR‐T cells were administered along with NY‐ESO‐1 Lips (equivalent to 50 μg of NY‐ESO‐1 peptides) or control substance, or NY‐ESO‐1 Lips by themselves, via intraperitoneal injection every 5 days, totaling two injections. Intraperitoneal administration of 250 μg of PD‐1 blockade occurred every 5 days with a total of two injections given. Mice were sacrificed on the 30th day, and the weight of tumor nodules was measured. Treatment in the mouse model began once tumor volumes reached around 50 mm^3^ subcutaneously. After 1 week, 10^7^ NY‐ESO‐1 TCR‐T cells along with NY‐ESO‐1 Lips (equal to 50 μg of NY‐ESO‐1 peptides) or control substances or NY‐ESO‐1 Lips by themselves were administered intravenously every 5 days for a total of two doses. Intravenous administration of PD‐1 blockades at a dose of 250 μg occurred every 5 days, with a total of two injections given. Tumor size was inspected every 3 days and calculated using the following formula:

$$\mathrm{Tumor}\mathrm{size}=\mathrm{length}\times \mathrm{widt}h^{2}\times 0.5$$

In safety research, key organs were collected, preserved in 4% paraformaldehyde, cut into sections, and then stained with H&E. The health status of the mice was monitored daily, and they were kept until any signs of illness appeared or the tumors grew larger than 30 mm in either length or width, at which time they were euthanized for animal welfare purposes.

### 4.14 Statistical analysis

All statistical analyses were conducted using GraphPad Prism V.7.0 (GraphPad Software). The groups compared statistically showed comparable variance. Sample size was not predetermined using statistical methods. Data are presented as mean ± SEM. The significance between groups was determined using the Student's *t*‐test. The threshold for statistical significance was established as *p* < 0.05.

## AUTHOR CONTRIBUTIONS

Qin Wang conducted experiments, wrote the main manuscript text, and prepared figures. Qin Wang, Fangcen Liu, Rui Peng, B.L., and Rutian Li conceived and designed all experiments. Fanyan Meng provided protocols and tools for research. QinWang, L.M., Jia Wei, X.H., and Shiyao Du assisted in conducting the experiments and preparing the figures. Lixia Yu, Wanmin Liu, Rutian Li, and Rui Peng assisted in manuscript preparation. Rutian Li and B.L. coordinated the research and verified the results. All authors reviewed the manuscript.

## CONFLICT OF INTEREST STATEMENT

The authors declare no conflicts of interest.

## ETHICS APPROVAL AND CONSENT TO PARTICIPATE

All animal experiments were performed in accordance with guidelines set by the Animal Care Committee at Drum Tower Hospital (Nanjing, China). The ethics committee of Drum Tower Hospital approved all experiments in this study (2019AE0152). All animal procedures were carried out in compliance with the guidelines. Investigators were not blinded for the animal studies. All efforts were made to minimize the number of animals used and their suffering. The blood collection procedure was carried out in accordance with the guidelines verified and approved by the Ethics Committee of Drum Tower Hospital. All donors signed an informed consent for scientific research statement (2021‐027‐02).

## Supporting information

Supporting Information

## Data Availability

Data are available upon reasonable request. All data relevant to the study are included in the article or uploaded as supplementary information. The data that support the findings of this study are available from the corresponding author upon reasonable request.
