# Supplementary material for: Liposome‐based in situ antigen‐modification strategy for “universal” T‐cell‐receptor engineered T cell in cancer immunotherapy
Source: MedComm (2020). 2024 Jul 7;5(7):e618. doi: 10.1002/mco2.618 (PMC11227616; doi:10.1002/mco2.618)
Supplement: Supplementary file 1 — Supporting Information [file MCO2-5-e618-s001.docx]

**
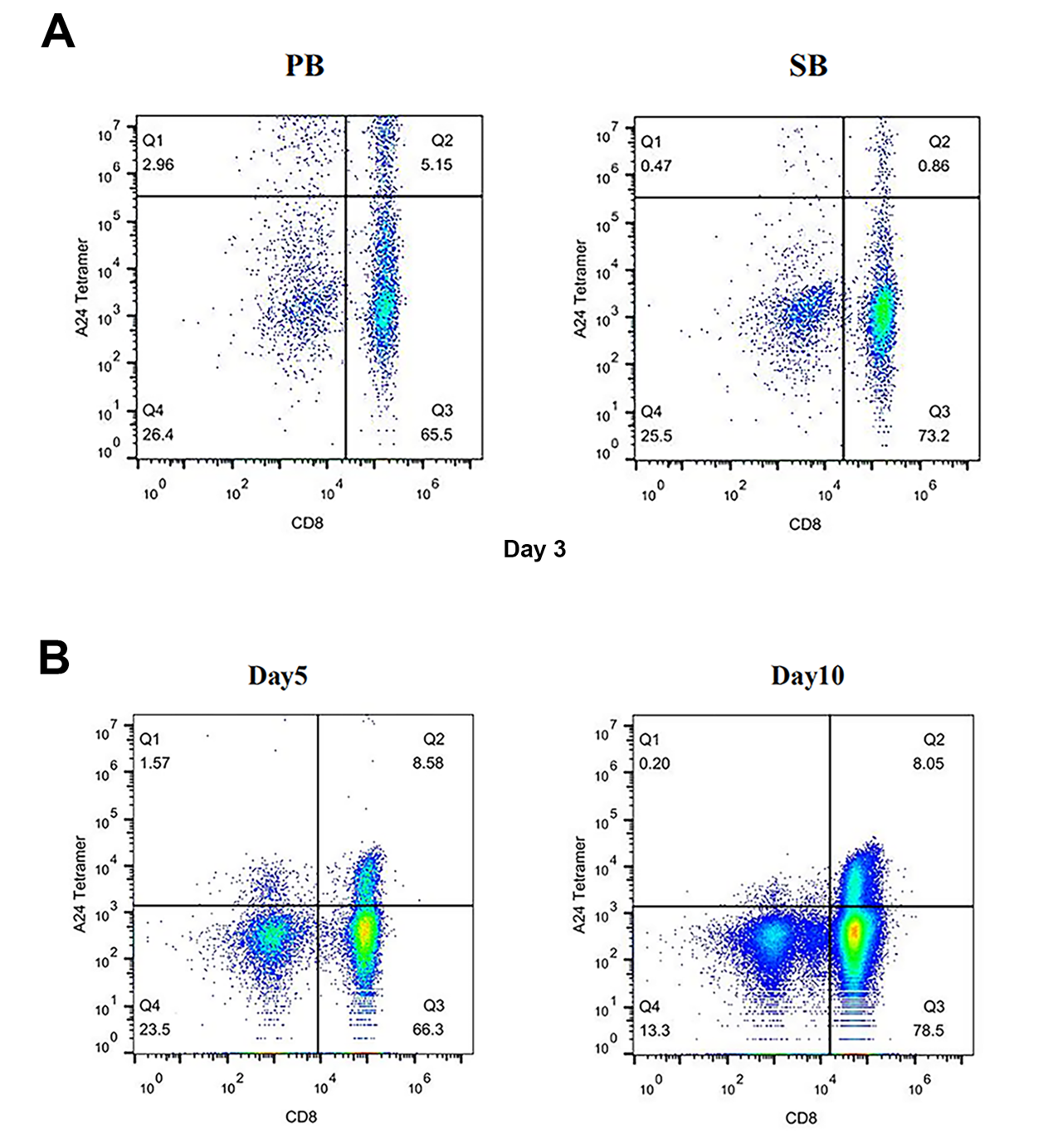
**

**Figure S1 Electric transfection efficacy of NY-ESO-1 TCR-T cells. (A) Transfection efficacy of PB VS SB electric transfection system to generate NY-ESO-1 TCR-T cells. (B) Expression stability of NY-ESO-1 TCR on PB electric transfection system transfected T cells.**

**
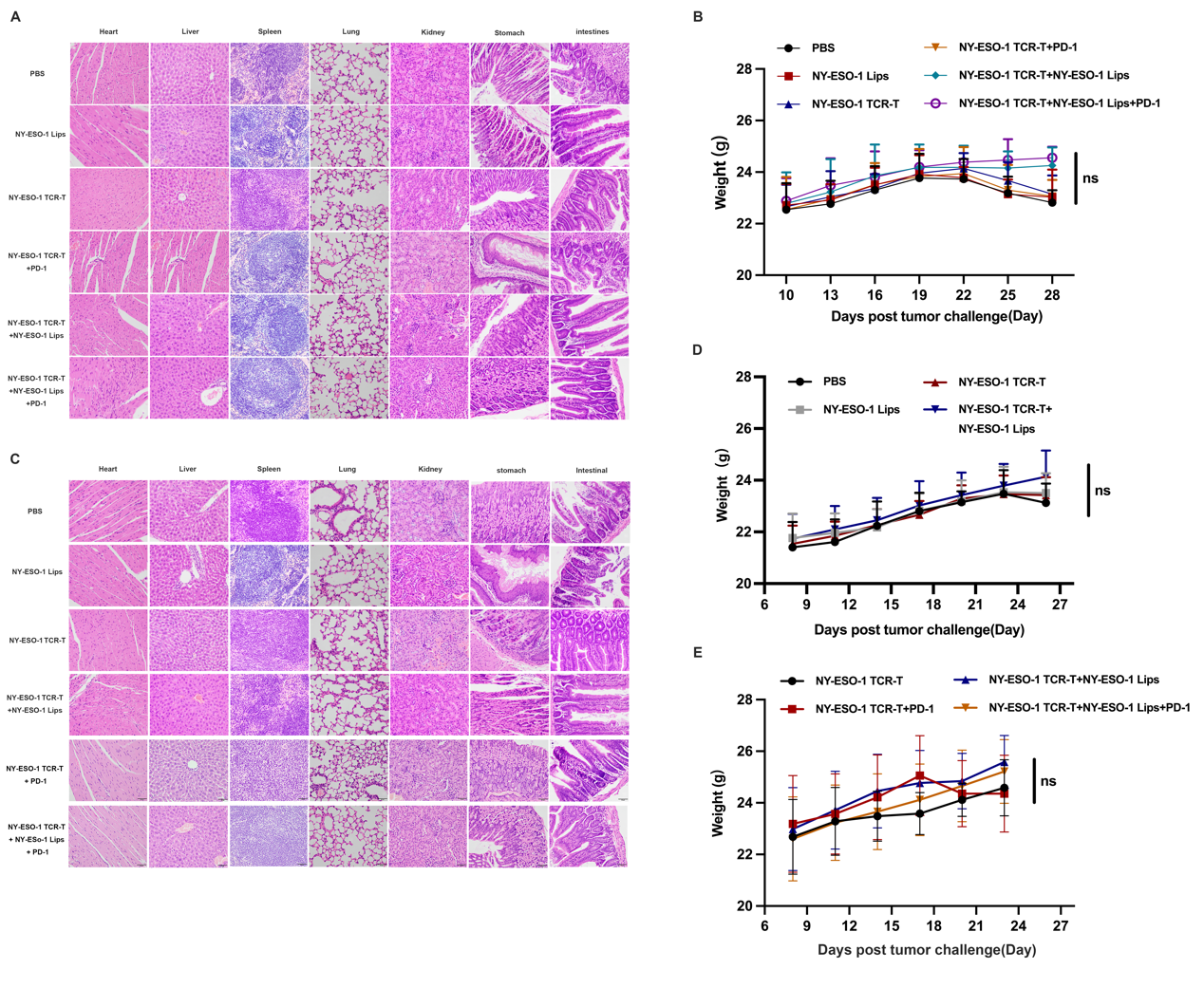
**

**Figure S2 Safety analysis of the experimental groups. (A-B) Alterations in body weight or H&E staining of lung, liver, heart, kidney, and spleen were observed in each treatment groups of MKN45-A2 peritoneal metastasis tumor model. (C-E) Alterations in body weight or H&E staining of lung, liver, heart, kidney, and spleen were observed in each treatment groups of MKN45-A2 subcutaneous mouse model.
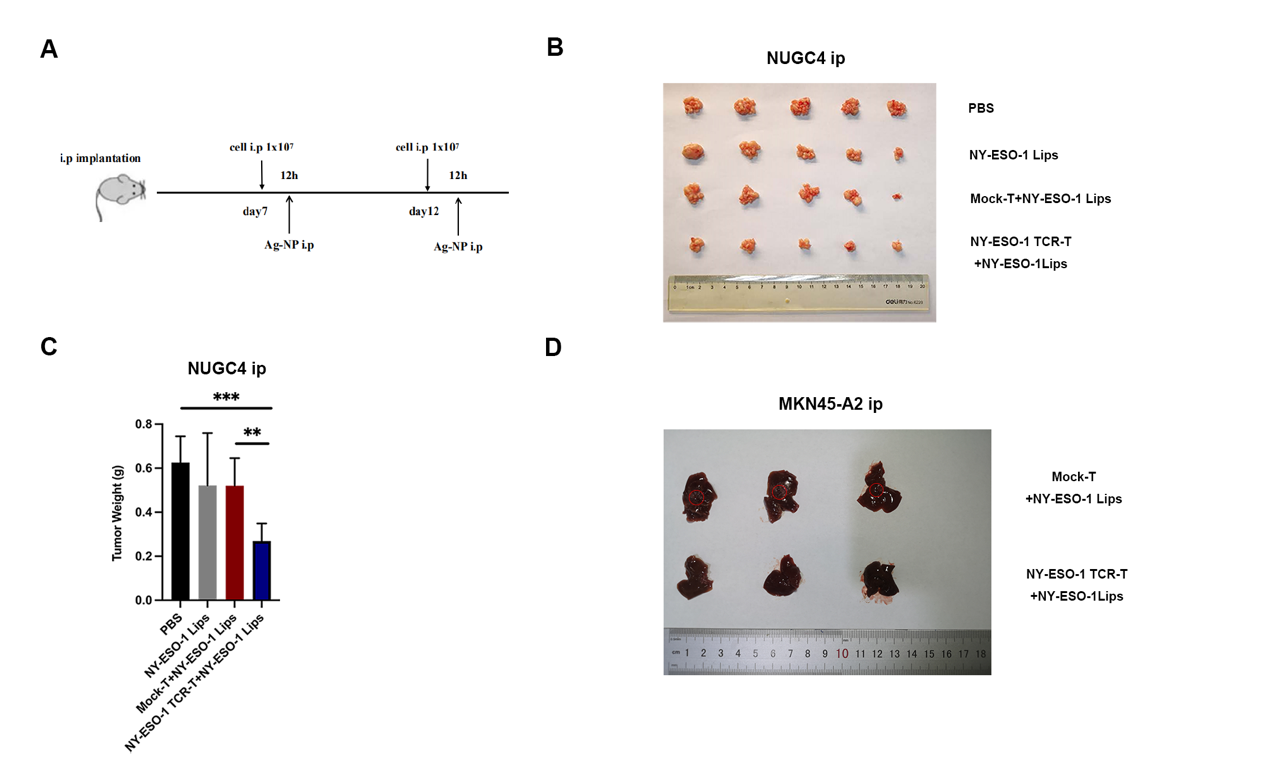
**

**Figure S3** **NUGC4 peritoneal metastasis tumor model, n=5. Seven days after tumor implantation (day 6), 1×10^7^ NY-ESO-1 Lips alone or combined with NY-ESO-1 TCR-T cells or Mock TCR-T cells were given intraperitoneally every five days for a total of two injections. Tumors were harvested after 16 days of treatment and weighed. (A)** **Schematic illustration of treatment process in peritoneal metastasis tumor model. (B) Tumor nodules in all treatment groups were photographed and (C) weighed in the NUGC4 peritoneal metastatic tumor model. Curves of tumor signal at three indicated timepoints during the treatment course. (D) Liver metastatic tumor nodules of MKN45-A2 peritoneal metastasis tumor models.**


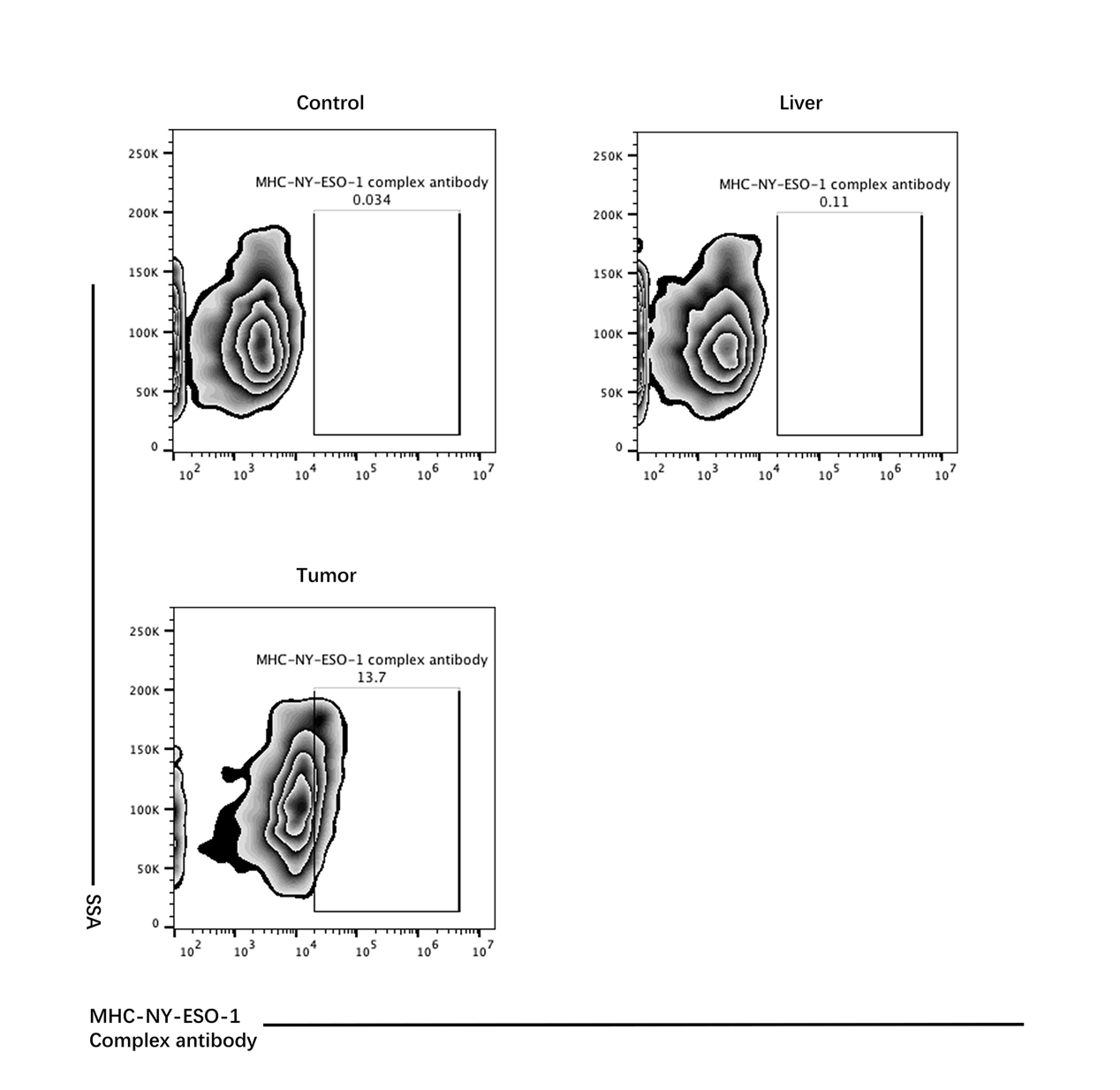


**Figure S4. Flow cytometry analysis of the proportion of liver cells with surface presentation of the MHC I-NY-ESO-1 peptide complex induced by the NY-ESO-1 Lips.**
